# Plant Responses to High Frequency Electromagnetic Fields

**DOI:** 10.1155/2016/1830262

**Published:** 2016-02-14

**Authors:** Alain Vian, Eric Davies, Michel Gendraud, Pierre Bonnet

**Affiliations:** ^1^Université d'Angers, Campus du Végétal, UMR 1345 IRHS, CS 60057, SFR 4207 QUASAV, 49071 Beaucouzé Cedex, France; ^2^Department of Plant and Microbial Biology, North Carolina State University, P.O. Box 7612, Raleigh, NC 27695, USA; ^3^Université Blaise Pascal, 24 avenue des Landais, 63177 Aubière Cedex, France; ^4^Institut Pascal, Université Blaise Pascal, BP 10448, 63000 Clermont-Ferrand, France; ^5^CNRS, UMR 6602, 63171 Aubière, France

## Abstract

High frequency nonionizing electromagnetic fields (HF-EMF) that are increasingly present in the environment constitute a genuine environmental stimulus able to evoke specific responses in plants that share many similarities with those observed after a stressful treatment. Plants constitute an outstanding model to study such interactions since their architecture (high surface area to volume ratio) optimizes their interaction with the environment. In the present review, after identifying the main exposure devices (transverse and gigahertz electromagnetic cells, wave guide, and mode stirred reverberating chamber) and general physics laws that govern EMF interactions with plants, we illustrate some of the observed responses after exposure to HF-EMF at the cellular, molecular, and whole plant scale. Indeed, numerous metabolic activities (reactive oxygen species metabolism, *α*- and *β*-amylase, Krebs cycle, pentose phosphate pathway, chlorophyll content, terpene emission, etc.) are modified, gene expression altered (calmodulin, calcium-dependent protein kinase, and proteinase inhibitor), and growth reduced (stem elongation and dry weight) after low power (i.e., nonthermal) HF-EMF exposure. These changes occur not only in the tissues directly exposed but also systemically in distant tissues. While the long-term impact of these metabolic changes remains largely unknown, we propose to consider nonionizing HF-EMF radiation as a noninjurious, genuine environmental factor that readily evokes changes in plant metabolism.

## 1. Introduction

High frequency electromagnetic fields (HF-EMF, i.e., frequencies from 300 MHz to 3 GHz, wavelengths from 1 m to 10 cm) are mainly human-produced, nonionizing electromagnetic radiations that do not naturally occur in the environment, excluding the low amplitude VHF (very high frequency) cosmic radiation. HF-EMF are increasingly present in the environment [1] because of the active development of wireless technology, including cell phones, Wi-Fi, and various kinds of connected devices. Since living material is not a perfect dielectric, it readily interferes with HF-EMF in a way that depends upon several parameters, including (but not restricted to) its general shape, the conductivity and density of the tissue, and the frequency and amplitude of the EMF. The interaction between the living material and the electromagnetic radiation may (or not) induce an elevation of the tissue temperature, thus defining the thermal (versus nonthermal) associated metabolic responses. In the case of a thermal response, the resulting heat dissipation is normalized with the specific absorption rate (SAR) index. This has led to considerable research efforts to study the possible biological effects due to exposure to HF-EMF. While the vast majority of these studies have focused on animals and humans because of health concerns, with contradictory or nonconclusive results [2], numerous experiments have also been performed on plants. Plants are outstanding models compared to animals to conduct such investigations: they are immobile and therefore keep a constant orientation in the EMF and their specific scheme of development (high surface area to volume ratio) makes them ideally suited to efficiently intercept EMF [3]. It is also quite easy in plants to achieve genetically stable plant lines through the selection of species that favor asexual reproduction [4] or self-pollination [5]. Furthermore, metabolic mutants are easily available for several species and constitute invaluable tools to understand the way the EMF signal is transduced [6]. Indeed, several reports have pointed out that plants actually perceive HF-EMF of even small amplitudes and transduce them into molecular responses and/or alterations of their developmental scheme [3–9]. The way that HF-EMF interact with plants remains essentially unanswered. However, since EMF evoke a multitude of responses in plants, they might be considered as a genuine environmental stimulus. Indeed, EMF exposure alters the activity of several enzymes, including those of reactive oxygen species (ROS) metabolism [7], a well-known marker of plant responses to various kinds of environmental factors. EMF exposure also evokes the expression of specific genes previously implicated in plant responses to wounding [5, 8] and modifies the development of plants [9]. Furthermore, these responses are systemic insofar as exposure of only a small region of a plant results in almost immediate molecular responses throughout the plant [6]. These responses were abolished in the presence of calcium chelators [6] or inhibitors of oxidative phosphorylation [10] which implies the involvement of ATP pools. In the present review, we describe exposure devices, SAR determination methods, and biological responses (at both the cellular/molecular and whole plant levels) observed after plant exposure to EMF. We focused this review on radiated (i.e., EMF that are emitted through an antenna) HF-EMF (mainly within the range of 300 MHz–3 GHz) and consequently will not address the biological effects of static magnetic fields (SMF), extremely low frequency electromagnetic fields (ELF), or HF current injection, since their inherent physical properties are dramatically different from those of high frequencies. Therefore, the HF-EMF we consider should be viewed through the prism of classical electromagnetism: macroscopic electrodynamics phenomena described in terms of vector and scalar fields.

## 2. Exposure Systems and Dosimetry

**Figure 1:**
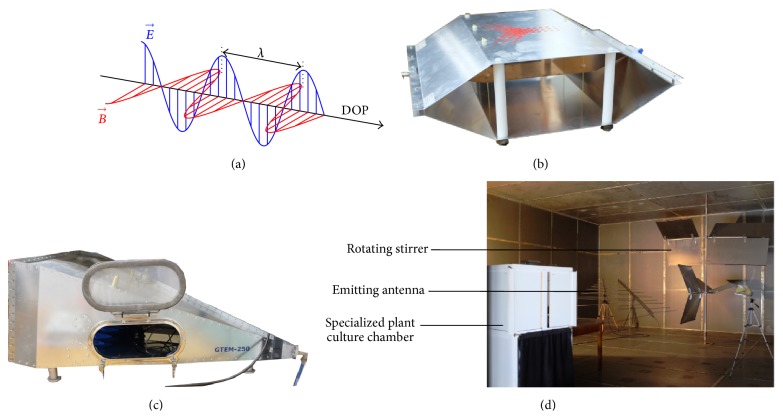
Electromagnetic wave and experimental set-up. (a) Schematic representation of an electromagnetic plane wave showing the transverse and space varying electric (*E*) and magnetic field (*B*). The wavelength (*λ*) is the distance between two crests. DOP: direction of propagation. (b) A TEM cell (transverse electromagnetic cell). (c) A GTEM cell (gigahertz transverse electromagnetic cell). (d) MSRC (mode stirred reverberation chamber). Note the double-sided metallic walls, the emitting antenna, the rotating stirrer, and the specialized culture chamber that stands in the “working volume” where the electromagnetic field characteristics have been extensively characterized.

HF-EMF are a combination of an electric field and a magnetic field governed by Maxwell's equations. At high frequency, these vector quantities are coupled and obey wave equations whether for propagating waves or for standing waves. In vacuum, the former travel at the speed of light (≈3 · 10^8^ m s^−1^) and have the structure of a plane wave (Figure 1(a)). In other media, the speed decreases and the spatial distribution for the electric and the magnetic fields are generally arbitrary (thus not being a plane wave). The latter, which do not propagate but vibrate up and down in place, appear in some particular conditions (e.g., bounded medium like metallic cavity) and play important roles in many physical applications (resonator, waveguide, etc.).

In both cases, HF-EMF are characterized by an amplitude of the electric (*E*) or magnetic (*H*) components (measured in volts or amperes per meter), a frequency *f* (number of cycles per second of the wave quantity, measured in hertz), and a wavelength *λ* (distance between wave crests, measured in meters). These properties are related through the following equation:

(1) $$\begin{array}{c} \lambda =\frac{c}{f}=c\times T, \end{array}$$

where *c* is the speed of the wave in the considered medium and *T* is the period of the wave (time between successive wave crests, measured in seconds). The wavelength *λ* is then the distance traveled by the wave during a period *T*.

The electromagnetic power density associated with an electromagnetic wave (measured in watts per square meter) is obtained by a vector product between the electric and magnetic field vectors (namely, the Poynting vector) for every point in space. The total HF-EMF power crossing any given surface is derived from Poynting's theorem [11]. For an incident plane wave in vacuum, the time-averaged electromagnetic power *P*_*i*_ (measured in watts) illuminating a surface of 1 m^2^ orthogonal to the direction of propagation is given by the following equation:

(2) $$\begin{array}{c} P_{i}=\frac{E^{2}}{2\times Z_{0}}, \end{array}$$

where *Z*_0_ is the characteristic impedance of free vacuum space (377 Ω).

The absorbed electromagnetic power (*Pd*), converted to heat by Joule effect in a volume (*V*) and averaged over a time period, is given by (3) for an electrically and magnetically linear material that obeys Ohm's law (conductivity *σ* in siemens per meter):

(3) $$\begin{array}{c} Pd={∭}_{v}^{}\frac{\sigma \times E^{2}}{2}dV. \end{array}$$

### 2.1. Diversity of Exposure Devices

Due to the wide variety of electromagnetic waves, physicians developed a lot of electromagnetic exposure facilities, mainly for electromagnetic compatibility (EMC) test purposes. Some of these devices are used for plant exposure to HF-EMF.

HF-EMF exposure set-up is usually made up with the following two basic elements: (i) HF source (radio frequency generator, Gunn oscillator) associated with a radiating element (antenna, strip-line) and (ii) a structure that allows the propagation of EM waves and the exposure of the sample. The simplest exposure set-up relies on the use of standard cell phones as a source of HF-EMF [12, 13] radiating in an open-area test site. While this apparatus has the advantage of being simple and economical, it poses many limitations that may compromise the quality of the exposure. Indeed, these communication devices are operated with different protocols that may modify or even interrupt the emitted power. Also, the biological samples are placed in the immediate neighborhood of the antenna, which is a region where the electromagnetic field is not completely established (near-field conditions) and therefore is difficult to measure; this situation may constitute an issue for bioelectromagnetics studies. These apparatuses are nowadays used only in a small proportion of studies. Moreover, the use of open-area test sites exposes the biological samples to the uncontrolled electromagnetic ambient environment. The use of shielded rooms is a good solution to overcome this issue. Indeed, anechoic chambers provide shielded enclosures, which are designed to completely absorb reflected electromagnetic waves. However, these facilities are often large structures requiring specific equipment and costly absorbers to generate an incident plane wave (far-field illumination) and are consequently seldom used for plant exposure [14, 15].

In contrast, numerous studies are based upon dedicated apparatus of relatively small volume (Figure 1(b)), namely, the transverse electromagnetic (TEM) cell [16]. TEM cells are usually quite small (about 50 cm long × 20 cm wide) and therefore only allow the use of seeds or seedlings as plant models. Many TEM cells are based upon the classic “Crawford cell” [17]. They consist of a section of rectangular coaxial transmission line tapered at each end to adapt to standard coaxial connectors. A uniform plane wave of fixed polarization and direction is generated in the sample space for experiments between the inner conductor (septum) and the upper metallic wall. Because this cost-efficient device is enclosed, high amplitude EMF can be developed with relatively little injected power. Under some conditions, two parallel walls of the TEM cell can be removed (therefore constituting the so-called open TEM cell) without dramatically compromising the performances. This configuration is adequate to allow plant lighting. Special attention must still be paid to the relative position of the samples in the system since the disposition of the different organs within the EMF could severely affect the efficiency of the plant samples' coupling with the electromagnetic field. The main TEM cell limitation is that the upper useful frequency is bound by its physical dimensions limiting the practical size of samples at high frequency.

The gigahertz transverse electromagnetic (GTEM) cell has emerged as a more recent EMF emission test facility (Figure 1(c)) [18]. It is a hybrid between an anechoic chamber and a TEM cell and could therefore be considered as a high frequency version of the TEM cell. The GTEM cell comprises only a tapered section, with one port and a broadband termination. This termination consists of a 50 Ω resistor board for low frequencies and pyramidal absorbers for high frequencies. This exposure device removes the inherent upper frequency limit of TEM cell while retaining some of its advantages (mainly the fact that no antenna set-up is required and the fact that high field strength could be achieved with low injected power).

Waveguides are another kind of screened enclosures that are seldom used in plant exposure [19, 20]. These classical and easy to use exposure devices generate traveling waves along the transmission coordinate and standing waves along the transverse coordinates. In contrast to the TEM cell, waveguides do not generate uniform plane waves but rather allow the propagation of more complex EMF, namely, propagation modes. Each mode is characterized by a cutoff frequency below which the mode cannot propagate. When the ends of the waveguide are short-circuited, a so-called resonant cavity is constituted, from which a recent large facility, originally designed for EMC studies, namely, the mode stirred reverberation chamber (MSRC, Figure 1(d)), is based. While this equipment is expensive and technically difficult to set up, it is the state of the art in terms of electromagnetic field characteristics, allowing the establishment of an isotropic and homogeneous field in a volume large enough to hold a dedicated plant culture chamber (either transparent or shielded toward EMF [6]). This latter characteristic permits experiments on large plants that are kept in an adequate controlled environment [6]. Our group pioneered the use of this facility, based on judicious combinations of standing waves patterns in a complex screened enclosure, in plant bioelectromagnetics studies [8] and extensively described the MSRC functionality [21]. Finally, each exposure set-up may differ in concept, polarization, frequency, or incident power but these setups always need to be optimally designed and based on well-understood physical concepts in order to assess well-controlled HF-EMF exposure conditions (homogeneity, repeatability, reproducibility, etc.).

### 2.2. Different Types of Exposure Signals

From each of the previous exposure devices, two very different types of EMF can be used to expose plants. The most commonly encountered mode is the continuous wave (CW) mode, in which the biological samples are continuously exposed for a specific duration to an EMF of given frequency and amplitude (rarely more than a few dozen V m^−1^). The second mode is the pulsed electromagnetic field (PEMF) mode, in which the biological samples are subjected to several series of discontinuous pulses of ultrashort duration EMF (within the range of *μ*s to ns) and usually of very high amplitude (up to several hundred kV m^−1^). This last kind of exposure [22, 23] is seldom used because of the scarcity and great complexity of the equipment needed to generate the EMF and the difficulty to design the dedicated antennae able to deliver such ultrashort power surges [24].

The HF-EMF could also be modulated (i.e., varied in time at a given, usually much lower frequency). Only a few studies explicitly addressed modulation effect on biological responses. Răcuciu et al. [25] exposed maize caryopses to low levels (7 dBm), 900 MHz RF field, for 24 h in either continuous wave (CW), amplitude modulated (AM), or frequency modulated (FM) modes. They found that 12-day-old plant lengths were reduced by about 25% in modulated EMF (AM or FM type) compared to control (unexposed samples), while CW exposure had an opposite (growth stimulation) effect, suggesting that EMF modulation actually modifies biological responses.

### 2.3. Dosimetry

In order to compare the biological effects observed in different exposure conditions, the National Council on Radiation Protection and Measurements officially introduced in 1981 an EMF exposure metric, the specific absorption rate (SAR). The formal definition of this basic dosimetry (the amount of dose absorbed) is “the time derivative of the incremental energy absorbed (*dW*) by (dissipated in) an incremental mass contained in a volume (*dV*) of a given density *ρ*.” From this definition and (3), the SAR (measured in W kg^−1^) is given by the following equation:

(4) $$\begin{array}{c} \text{SAR}=\frac{d}{dt}\left(\;\frac{dW}{\rho \times dV}\;\right)=\frac{\sigma \times E^{2}}{2\times \rho }. \end{array}$$

SAR is the power absorbed by living tissue during exposure to CW-EMF (this quantity does not apply to PEMF mode because of the very short duration of the pulses that do not cause temperature increase in the samples). SAR can be calculated from the dielectric characteristics of plant tissues at the working frequencies, using (4). While *ρ* could be easily determined, the value of *σ* is dependent upon the frequency and is difficult to assess in the range of GHz. It is usually evaluated from the literature [40], since the experimental set-up to measure this parameter at a given frequency (waveguide, open waveguide, and coaxial line technique, e.g., D-Line) is rarely used because of its complex set-up. From the biological heat-transfer equation, the SAR can also be determined using the temperature increase evoked in plant tissue after exposure to EMF, using the following equation:

(5) $$\begin{array}{c} \text{SAR}=C\times {\left(\;\frac{dT}{dt}\;\right)}_{t\to 0}, \end{array}$$

where *C* is the heat capacity (J K^−1^ kg^−1^, which is available for some tissues in the literature) and *dT* (measured in Kelvin) is the sample temperature increase corresponding to the elapsed time *dt* (measured in second) since the beginning of HF-EMF exposure. Either for animals or plants, the SAR measurement is subject to uncertainty [46]. Since the specific heat is frequency independent and the temperature distribution is usually more uniform than the internal electric field, (5) provides, for detectable temperature increases, a better way for SAR estimation.

In animal and human tissue, SAR is determined using dedicated phantoms [47] filled with a special liquid that mimics the dielectric properties of biological fluids. While this approach is adequate in animals, in which the developmental scheme produced volumes, it could not be adapted to most plant organs (e.g., leaves) that have a high surface area to volume ratio [3] but could be used in fruits and tuberous structures. In contrast, surface temperature can be easily assessed with dedicated instruments (e.g., Luxtron® fiber optic temperature probe) and used to feed (5) [45]. The SAR can also be determined using the differential power method based on the measurement of power absorption (reviewed in [48]) that takes place in the absence or presence of biological samples [39]. The SAR is then calculated by dividing the absorbed power by the mass of the living material.

## 3. Biological Responses

Biological responses should be considered as reporters of, and evidence for, the plant's ability to perceive and interact with EMF. These responses can take place at the subcellular level, implying molecular events or modification of enzymatic activities, or at the level of the whole plant, taking the form of growth modification. Tables 1–3 summarize some work reporting HF-EMF effects observed at the scale of the whole plant, biochemical processes, or gene regulation, respectively.

### 3.1. Cellular and Molecular Level

**Figure 2:**
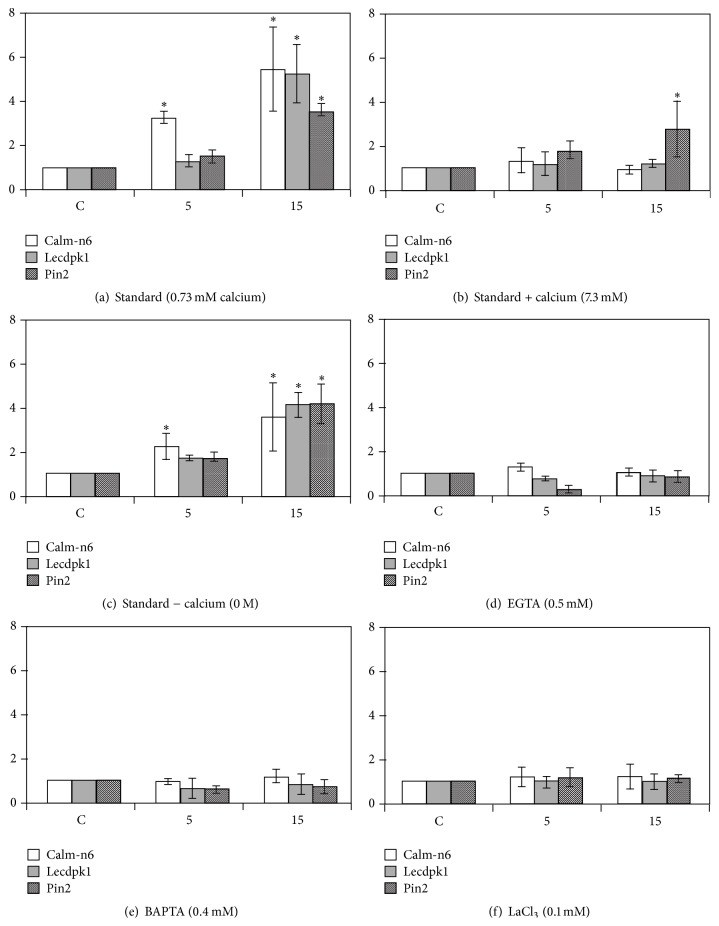
Effect of calcium concentration in culture medium on calm-n6 (calmodulin), pin2 (proteinase inhibitor), and lecdpk1 (calcium-dependent protein kinase) transcript accumulation in response to the HF-EMF exposure. (a) Standard medium (0.73 mM of calcium). (b) Tenfold extra calcium (7.3 mM). (c) No calcium (0 mM). (d) No calcium (0 mM) with 0.5 mM of EGTA. (e) No calcium (0 mM) with 0.4 mM of BAPTA (1,2-bis(o-aminophenoxy)ethane-N,N,N′,N′-tetraacetic acid), a specific Ca^2+^ chelator. (f) No calcium (0 mM) with 0.1 mM LaCl_3_. Bars represent mean values ± SE from at least three independent experiments. An asterisk over the bars states the significant differences according to the one-sided Mann-Whitney *U* test. Reproduced from [10], with permission.

**Figure 3:**
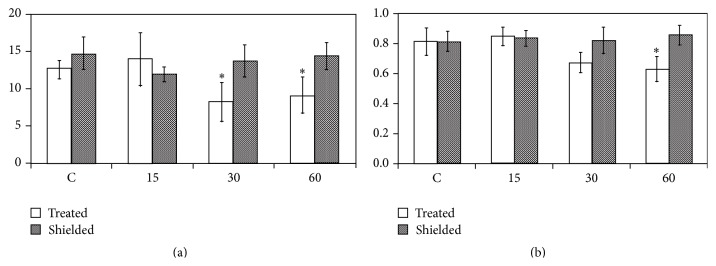
ATP concentration and adenylate energy charge (AEC) changes after HF-EMF exposure (5 V m^−1^, 10 min) in a mode stirred reverberation chamber. C: control, unexposed plants. 15, 30, and 60: time (min) after the end of HF-EMF exposure. (a) ATP concentration (pmol mg^−1^ Prot.). (b) Adenylate energy charge (ratio). Bars represent mean values ± SE from at least three independent experiments. An asterisk over the bars states the significant differences according to the one-sided Mann-Whitney *U* test. Reproduced from [10], with permission.

**Figure 4:**
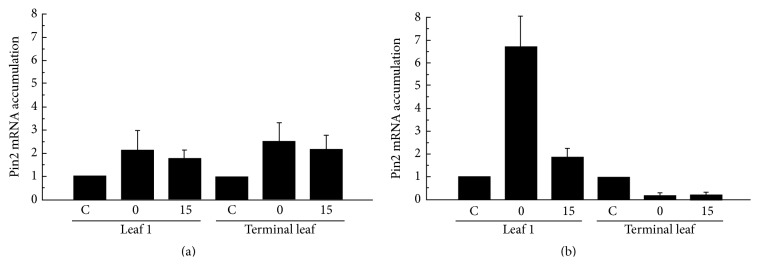
Local and systemic responses after HF-EMF exposure. (a) Systemic response after local exposure to HF-EMF in wild type. Local and distant responses after stimulation of leaf 1 (with the rest of the plant being protected from the EMF). The stimulated tissue (leaf 1) and distant one (terminal leaf) both displayed responses (accumulation of pin2 transcript). HF-EMF exposure: 5 V m^−1^, 10 min. (b) Impairment of distant response after exposure to HF-EMF in* sitiens *(ABA deficient) mutant. The stimulated tissue (leaf 1) displays the response to EMF exposure (accumulation of pin2 transcript), while the response in the distant tissue (terminal leaf) is impaired. HF-EMF exposure: 5 V m^−1^, 10 min. Reproduced from [6], with permission.

Numerous reports [4, 7, 33] indicate an increase in the production of malondialdehyde (MDA, a well-known marker of membrane alteration) along with ROS metabolism activation after exposing plants to HF-EMF (Table 1). Membrane alteration and ROS metabolism activation are likely to establish transduction cascades that enable specific responses. Indeed, the critical role of calcium, a crucial second messenger in plants, has long been pointed out [6, 10]: the responses (e.g., changes in calm-n6, lecdpk-1, and pin2 gene expression) to EMF exposure are severely reduced when plants are cultivated with excess of calcium or in the presence of calcium counteracting agents (Figure 2) such as chelators (EGTA and BAPTA) or a channel blocker (LaCl_3_). The importance of calcium in the establishment of the plant response is also highlighted by the fact that early gene expression associated with EMF exposure involves at least 2 calcium-related products (calmodulin and calcium-dependent protein kinase) [5, 10]. This response is also energy-dependent: an important drop (30%, Figure 3) in ATP content and adenylate energy charge (AEC) occurs after HF-EMF exposure [10]. It is not clear for now if the AEC drop is the consequence of altered membranes allowing passive ATP exit or if higher consumption of ATP occurred because of increased metabolic activity. Indeed, it is well known that a drop in AEC stimulates the catabolic enzymatic pathways through allosteric modulations. Nevertheless, inhibiting ATP biosynthesis with the decoupling agent carbonyl cyanide* m*-chlorophenyl hydrazone (CCCP) abolished plant responses to EMF exposure [10]. Nitric oxide (NO) is another signaling molecule that is tightly related to environmental factors' impact on plants [49]. NO rapidly increases after various kinds of stimuli including drought stress or wounding. Chen et al. [50] recently demonstrated the increased activity of nitric oxide synthase and accumulation of NO after exposing caryopses of wheat for 10 s to high power 2.45 GHz EMF. Similarly, Qiu et al. [51] showed in wheat that the tolerance to cadmium evoked by microwave pretreatment was abolished by the addition of 2-(4-carboxyphenyl)-4,4,5,5-tetramethylimidazoline-1-oxyl-3-oxide (carboxy-PTIO), an NO scavenger, suggesting that microwave-induced NO production was involved in this mechanism. Taken together, these results advocate for the EMF induction of NO synthase. However, these studies used high power EMF (modified microwave oven) as stimulating tool and the fact that a temperature increase of the sample was the cause of NO increase is not excluded. To our knowledge, the involvement of NO has not yet been demonstrated after low power (i.e., nonthermal) EMF exposure. Furthermore, well-known actors of plant responses to environmental stimuli are also involved: the tomato mutants* sitiens* and* JL-5 *for abscisic (ABA) or jasmonic (JA) acids biosynthesis, respectively, display normal responses (accumulation of stress-related transcripts) when whole plants are exposed to EMF [6]. In contrast, very rapid distant responses to local exposure that occur in the wild plants (Figure 4(a)) are impaired in* sitiens* ABA mutant (Figure 4(b)) and* JL-5 *mutants, highlighting the existence of a transmitted signal (whose genesis and/or transmission is dependent on ABA and JA) in the whole plant after local exposure [6]. The nature of this signal is still unknown, but very recent work has demonstrated that membrane potential is affected after exposure to EMF [14]. It could therefore be hypothesized that electrical signals (action potential and/or variation potential) could be the transmitted signal, strongly implying that HF-EMF is a genuine environmental factor.

#### 3.1.1. Alterations of Enzymatic Activities

Table 1 summarizes some of the enzymatic activities that are modified after exposing plants to HF-EMF. As previously noted, ROS metabolism is very often activated after plant exposure to EMF. Enzymatic activities such as peroxidase, catalase, superoxide dismutase, and ascorbate peroxidase have twofold to fourfold increase [4, 7, 18, 27, 33]. The question remains open to determine if this could be the consequence of a direct action of EMF on living tissue. Indeed, the very low energy that is associated with the EMF at these frequencies makes them nonionizing radiations. Side effects of elevated ROS metabolism are also noted: H_2_O_2_ production [4, 7], MDA increases [4, 7, 33], and protein damage [30]. An increase in polyphenol oxidase [27] and phenylalanine ammonia-lyase [26] may indicate stress responses linked to an increased lignification, a common response of plants to environmental stress.

Protein content is reduced in* Vigna *and* Phaseolus* [27, 32] as well as in* Triticum* [13]. It is not yet known if the decrease in protein content results from an increase in protein degradation and/or a decrease in protein synthesis, but this may constitute a stimulating field of investigation, since evidence shows that mRNA selection from translation occurs after plant exposure to HF-EMF [10]. Hydrolytic enzymatic activities (*α*- and *β*-amylases and invertases) responsible for the production of soluble sugar increase in germinating seeds after exposure to HF-EMF [12, 28, 32], while the starch phosphorylase activity, phosphorolytic and potentially reversible, is diminished. In contrast, HF-EMF exposure causes a drop of soluble sugar that may be related to the inhibition of Krebs cycle and pentose phosphate pathway in* Plectranthus* (Lamiaceae) leaves after exposure to 900 MHz EMF [29], suggesting that seeds and adult leaves respond in a different way to HF-EMF exposure. The accumulation of proline, reported by several authors [7, 33], and an increase in terpenoid emission and content in aromatic plants [34] are also classical responses of plants to environmental stresses.

#### 3.1.2. Modification of Gene Expression

While numerous reports focused on enzymatic activities alterations after exposure to EMF, only a few studies concentrate on gene expression modifications (Table 2). Tafforeau et al. [44] demonstrated using Gunn generator (105 GHz) several reproducible variations in 2D gel electrophoresis profiles, showing that gene expression is likely to be altered by the exposure treatment. Jangid et al. [52] provided indirect proof (RAPD profiles) suggesting that high power microwave irradiation (2450 MHz, 800 W cm^−2^) modifies gene expression in* Vigna aconitifolia*, while these results do not exclude a possible thermal effect of microwave treatment.* Arabidopsis thaliana *suspension-cultured cells exposed to HF-EMF (1.9 GHz, 8 mW cm^−2^) showed differential expression of several genes (*p* values < 0.05) compared to the control (unexposed) condition in microarray analysis [36]. Most of them are downregulated (while At4g39675, At5g10040, and AtCg00120 displayed a slight increase; see Table 2). However, the RT-PCR *p* value lowers the significance of these variations and these authors consequently concluded the absence of HF-EMF effect on plant gene expression. In contrast, short duration, high frequency, low amplitude EMF exposure (10 min, 900 MHz, 5 V m^−1^) performed on whole 3-week-old tomatoes in MSRC [5, 6, 8, 10] demonstrated altered expressions of at least 5 stress-related genes (Table 2), suggesting that whole plants are more sensitive to HF-EMF than cultured cells. These experiments have been independently replicated by Rammal et al. [35], using a longer exposure period and a far less sophisticated exposure set-up (cell phone). Stress responses of plants quite often display a biphasic pattern [53]: a very rapid increase in transcript accumulation that lasts 15–30 min, followed by a brief return to basal level, and then a second increase (after 60 min). This pattern was observed after tomato exposure to EMF so we questioned the meaning of the early and late population of transcripts in terms of physiological significance by measuring their association to polysomes (which reflects their putative translation to proteins). We found that the early (0–15 min) mRNA population was only faintly associated with polysomes, yet being poorly translated, while the late mRNA population (60 min) is highly associated with polysomes [10]. This result strongly suggests that only the late mRNA population may have a physiological importance since it is the only one to be efficiently translated into proteins.

### 3.2. Whole Plant Level

The biochemical and molecular modifications observed after plant exposure to EMF and described in the previous paragraphs might induce morphogenetic alterations of plant development. Indeed, an increasing number of studies report modifications of plant growth after exposure to HF-EMF (Table 3). These treatments are effective at different stages of plant development (seeds, seedlings, or whole plants) and may affect different organs or developmental processes including seeds germination and stem and root growth, indicating that biological samples of even small sizes (a few mm) are able to perceive HF-EMF. Seed exposure to EMF generally results in a reduced germination rate [27, 37, 39], while in other cases germination is unaffected [42] or even stimulated [16]. The seedlings issued from EMF-exposed seeds displayed reduced growth of roots and/or stem [13, 28, 32, 37–39, 41] but rarely a stimulatory effect [16]. This point strongly differs from exposure to static magnetic fields or extremely low frequency EMF, in which the stimulatory effects on growth are largely predominant [54]. Ultrashort pulsed high power EMF (PEMF, 4 *μ*s, 9.3 GHz, 320 kV m^−1^) also tends to stimulate germination of seeds of radish, carrot, and tomato and increase plant height and photosynthetic surface area in radish and tomato [20] and roots of tobacco seedlings [22]. These different effects of PEMF compared to HF-EMF on plants may be related to their fundamental difference in terms of physical properties. Exposure to HF-EMF of seedlings or plants (rather than seeds) also generally resulted in growth inhibition [9, 18, 27, 28, 39]. Singh et al. [7] showed that rhizogenesis (root number and length) is severely affected in mung bean after exposure to cell phone radiation, possibly through the activation of several stress-related enzymes (peroxidases and polyphenol oxidases). Akbal et al. [38] showed that root growth was reduced by almost 60% in* Lens culinaris* seeds exposed in the dormant state to 1800 MHz EMF radiation. Concomitantly, these authors reported an increase in ROS-related enzymes, lipid peroxidation, and proline accumulation, with all of these responses being characteristic of plant responses to stressful conditions. Afzal and Mansoor [13] investigated the effect of a 72 h cell phone exposure (900 MHz) on both monocotyledonous (wheat) and dicotyledonous (mung bean) plants seeds: germination was not affected, while the seedlings of both species displayed growth inhibition, protein content reduction, and strong increase in the enzymatic activities of ROS metabolism. It is however worth noting that growth of mung bean and water convolvulus seedlings exposed at a lower frequency (425 MHz, 2 h, 1 mW) is stimulated because of higher elongation of primary root [11], while duckweed (*Lemna minor*, Araceae) growth was significantly slowed down not only by exposure at a similar frequency (400 MHz, 4 h, 23 V m^−1^) but also after exposure at 900 and 1900 MHz for different field amplitudes (23, 41, and 390 V m^−1^) at least in the first days following the exposure [18]. Surducan et al. [15] also found stimulation of seedling growth in bean and maize after exposure to EMF (2.452 GHz, 0.005 mW cm^−2^). Senavirathna et al. [55] studied real-time impact of EMF radiation (2 GHz, 1.42 W m^−2^) on instantaneous growth in the aquatic plant, parrot's feather (*Myriophyllum aquaticum*, Haloragaceae), using nanometer scale elongation rate fluctuations. These authors demonstrated that EMF-exposed plants displayed reduced fluctuation rates that lasted for several hours after the exposure, strongly suggesting that plants' metabolism experienced a stressful situation. It is worth noting that the exposure did not cause any plant heating (as measured using sensitive thermal imaging). Some other kind of morphological changes also occurred after plant exposure to HF-EMF: induction of epidermal meristems in flax [44], flower bud abscission [43], nitrogen-fixation nodule number increase in leguminous [42], or delayed reduced growth of secondary axis in* Rosa* [45].

These growth reductions may be related to a lower photosynthetic potential since Răcuciu et al. [40] showed that exposing 12-day-old maize seedlings to 0.47 W kg^−1^ 1 GHz EMF induces a drop in photosynthetic pigment content: the diminution was especially important in chlorophyll a, which was reduced by 80% after 7 h of exposure. Ursache et al. [56] showed that exposure of maize seedlings to microwave (1 mW cm^−2^, 10.75 GHz) also caused a drop in chlorophyll a and b content. Similarly, Hamada [57] found a decrease in chlorophyll content in 14-day-old seedlings after exposing the caryopses for 75 min at 10.5 GHz. Kumar et al. showed a 13% decrease in total chlorophyll after 4 h exposure of maize seedlings to 1800 MHz (332 mW m^−2^). These modifications may be related to abnormal photosynthetic activity, which relies on many parameters, including chlorophyll and carotenoid content. Senavirathna et al. [58] showed that exposing duckweeds to 2–8 GHz, 45–50 V m^−1^ EMF induced changes in the nonphotosynthetic quenching, indicating a potential stressful condition. Three aromatic species belonging to Apiaceae family (*Petroselinum crispum*,* Apium graveolens*,* and Anethum graveolens*) strongly respond to global system for mobile communications radiation (GSM, 0.9 GHz, 100 mW cm^−2^) or wireless local area network (WLAN, 2.45 GHz, 70 mW cm^−2^) exposure by decreasing the net assimilation rate (over 50%) and the stomatal conductance (20–30%) [34].

## 4. Conclusion and Future Prospects

An increasing number of reports highlight biological responses of plants after exposure to HF-EMF at the molecular and the whole plant level. The exposure conditions are, however, far from being standardized and illustrate the diversity of exposure conditions employed. However, future work should avoid exposure in near-field conditions (i.e., in immediate vicinity of the emission antenna) where the field is instable and difficult to characterize. Similarly, the use of communication devices (i.e., cell phones) should be avoided as emission sources since it may be difficult to readily control the exposure conditions because of built-in automation that may overcome the experimental set-up. The use of specialized devices (TEM cells, GTEM cell, waveguides, MSRC, etc.) in which a precise control of exposure condition can be achieved is highly preferable.

Shckorbatov [59] recently reviewed the possible interactions mechanisms of EMF with living organisms. While the classical targets (interaction with membranes, free radicals, and intracellular regulatory systems) have all been observed in plants, a convincing interpretation of the precise mechanism of HF-EMF interaction with living material is still needed. Alternative explanation (i.e., electromagnetic resonance achieved after extremely high frequency stimulation which matches some kind of organ architecture) has also been proposed for very high frequency EMF (several dozen GHz) [60]. However, the reality of this phenomenon* in vivo* (studied for now only through numerical simulations) and its formal contribution to the regulation of plant development have not yet been experimentally established. Amat et al. [61] proposed that light effects on plants arose not only through chromophores, but also through alternating electric fields which are induced in the medium and able to interact with polar structures through dipole transitions. The possible associated targets (ATP/ADP ratio, ATP synthesis, and Ca^2+^ regulation) are also those affected by exposure to HF-EMF [10]. It could therefore be speculated that HF-EMF may use similar mechanisms. The targeted pathways, especially Ca^2+^ metabolism, are well known to modulate numerous responses of plants to environmental stress. While deeper understanding of plant responses to HF-EMF is still needed, these treatments may initiate a set of molecular responses that may affect plant resistance to environmental stresses, as already demonstrated in wheat for CaCl_2_ [62] or UV [63] tolerances, and constitute a valuable strategy to increase plant resistance to environmental stressful conditions.

## Figures and Tables

**Table 1:** Metabolic pathways affected after plant exposure to HF-EMF radiations.

Enzymes or metabolites	Metabolic pathways	Organisms	Exposure conditions	Response to EMF
Phenylalanine ammonia-lyase	Phenylpropanoids	*Phaseolus vulgaris*	N/A (PEMF)	Synergistic action with growth regulators in cultured cells [26]

Polyphenol oxidase	Polyphenols	*Vigna radiata*	900 MHz, up to 4 h, 8.55 *μ*W cm^−2^	8.5-fold increase [27]

*α*- and *β*-amylases	Starch metabolism	*Vigna radiata*	900 MHz, up to 4 h, 8.55 *μ*W cm^−2^	2.5- and 15-fold increase for *α*- and *β*-amylases, respectively [27]

*α*- and *β*-amylases, starch phosphorylases	Starch metabolism	*Zea mays*	1800 MHz, up to 4 h, 332 mW m^−2^	2-fold increase for amylases. −73% for starch phosphorylases [28]

Water soluble sugars	Sugar metabolism	*Phaseolus vulgaris*	900 MHz, 4 h	2-fold reduction in soluble sugars [12]

Acid and alkaline invertases	Sucrose metabolism	*Zea mays*	1800 MHz, up to 4 h, 332 mW m^−2^	1.8- and 2.6-fold increase for acid and alkaline forms, respectively [28]

Malate and NADP isocitrate dehydrogenases, glucose-6P dehydrogenase	Krebs cycle, pentose phosphate pathway	*Plectranthus*	900 MHz, 1 h	Lower activity (−10 to −30%) at the end of the stimulus and then a 2-fold increase 24 h later [29]

ATP content and adenylate energy charge (AEC)	Energetic metabolism	*Solanum lycopersicon*	900 MHz, 10 min, 5 V m^−1^	Drop of ATP content (30%) and AEC (0.8 to 0.6) 30 min after the stimulus [10]

MDA content, H_2_O_2_, superoxide dismutase, catalase, guaiacol peroxidase, glutathione reductase, ascorbate peroxidase	Lipid peroxidation-oxidative metabolism	*Vigna radiata*	900 MHz, 8.55 *μ*W cm^−2^	All oxidative metabolism markers increased (2-fold to 5-fold) [7]

MDA and H_2_O_2_ content, catalase, ascorbate peroxidase	Lipid peroxidation	*Lemna minor*	400 and 900 MHz, 2 to 4 h, 10 to 120 V m^−1^	MDA and H_2_O_2_ content, catalase and ascorbate peroxidase activities increased (10–30%) [30]

Peroxidases	Oxidative metabolism	*Vigna radiata, Lemna minor*	900 MHz, 1 to 4 h, 8.55 *μ*W cm^−2^ or 41 V m^−1^	Peroxidase activities increased [18, 27]

MDA, oxidized and reduced glutathione, NO synthase	Oxidative metabolism-NO metabolism	*Triticum aestivum*	2.45 GHz, 5 to 25 s, 126 mW mm^−2^ concomitantly with NaCl treatment	Exposure to EMF reduced the oxidative response of plants to high salt treatment [31]

Protein metabolism-DNA damage	Oxidative protein and DNA damage (comet assay)	*Nicotiana tabacum*	900 MHz, 23 V m^−1^	Carbonyl content and tail DNA value increased (1.8-fold and 30%, resp.) [30]

Protein metabolism	Protein content	*Phaseolus vulgaris, Vigna radiata, Triticum aestivum*	Cell phone, 4 h	Drop in protein content in *Phaseolus* (71%) and *Vigna* (57%) [27, 32] and *Triticum* [13]

Amino acid metabolism	Proline accumulation	*Zea mays, Vigna radiata*	940 MHz, 2 days Cell phone, 2 h, 8.55 *μ*W cm^−2^	1.8- and 5-fold increase in *Z. mays* [33] and *V. radiata* [7], respectively

Global terpene emission	Monoterpene metabolism	*Petroselinum crispum, Apium graveolens, Anethum graveolens*	900–2400 MHz, 70–100 mW m^−2^	Enhanced emission of terpene compounds [34]

**Table 2:** Genes whose expression is altered after plant exposure to HF-EMF.

Gene	Organism	Function	Exposure conditions	Response to EMF exposure
lebZIP1	*Solanum lycopersicon, whole plant*	Transcription factor	900 MHz, 5 V m^−1^, CW in a MSRC	Increase (3-fold to 4-fold) [6, 8]

lebZIP1	*Solanum lycopersicon, whole plant*	Transcription factor	Cell phone	Increase (3-4-fold) [35]

cam	*Solanum lycopersicon, whole plant*	Ca^2+^ signal transduction	900 MHz, 5 V m^−1^, CW in a MSRC	Increase (5-fold) [5, 10]

cdpk	*Solanum lycopersicon, whole plant*	Ca^2+^ signal transduction	900 MHz, 5 V m^−1^, CW in a MSRC	Increase (5-fold) [10]

cmbp	*Solanum lycopersicon, whole plant*	mRNA metabolism	900 MHz, 5 V m^−1^, CW in a MSRC	Increase (6-fold) [5]

pin2	*Solanum lycopersicon, whole plant*	Proteinase inhibitor	900 MHz, 5 V m^−1^, CW in a MSRC	Increase (4.5-fold [5] and 2.5-fold) [6]

pin2	*Solanum lycopersicon, whole plant*	Proteinase inhibitor	900 MHz, cell phone	Increase (2-fold) [35]

At4g26260	*Arabidopsis thaliana, cell suspension culture*	Similar to myo-inositol oxygenase	1.9 GHz, 8 mW cm^−2^	Decrease (0.3-fold) [36]

At3g47340	*Arabidopsis thaliana, cell suspension culture*	Glutamine-dependent asparagine synthetase	1.9 GHz, 8 mW cm^−2^	Decrease (0.4-fold) [36]

At3g15460	*Arabidopsis thaliana, cell suspension culture*	Brix domain protein	1.9 GHz, 8 mW cm^−2^	Decrease (0.5-fold) [36]

At4g39675	*Arabidopsis thaliana, cell suspension culture*	Expressed protein	1.9 GHz, 8 mW cm^−2^	Increase (1.5-fold) [36]

At5g10040	*Arabidopsis thaliana, cell suspension culture*	Expressed protein	1.9 GHz, 8 mW cm^−2^	Increase (1.4-fold) [36]

AtCg00120	*Arabidopsis thaliana, cell suspension culture*	ATPase alpha subunit (chloroplast)	1.9 GHz, 8 mW cm^−2^	Increase (1.4-fold) [36]

**Table 3:** Morphogenetic responses observed after plant exposure to HF-EMF.

Plant species	Exposure conditions	Responses to HF-EMF exposure and references
*Raphanus sativus*	Gunn generator 10.5 GHz, 14 mW, exposure of seeds and hypocotyls	Germination inhibition (45%), reduction of hypocotyl elongation (40%) [37]

*Lens culinaris*	Cell phone, 1800 MHz (1 mW), exposure of dormant seeds	Reduction of seedlings' root growth (60%) and mitotic index (12%). Abnormal mitosis increased (52%) [38]

*Vigna radiata*	Cell phone, 900 MHz, 8.55 *μ*W cm^−2^	Rhizogenesis (root number and length) severely affected [7]

*Vigna radiata*	Cell phone, 900 MHz, 8.55 *μ*W cm^−2^	Inhibition of germination (50%), hypocotyl (46%), and root growth (59%). Dry weight reduced by 43% [27]

*Phaseolus aureus, Vigna radiata*	Cell phone, 4 h exposure	Root and stem elongations severely affected (−44 and −39%, resp.) [12, 32]

*Vigna radiata, Lablab purpureus*	1.8 GHz, 0.48–1.45 mW cm^−2^	Reduction of height and fresh weight [39]

*Zea mays*	1 GHz, 1 to 8 h, 0.47 W cm^−2^	Reduced growth of 12-day-old plants (about 50% after 8 h of exposure) [40]

*Zea mays*	1800 MHz, 4 h, 332 mW m^−2^	Reduced growth of roots and coleoptiles (16 and 22%, resp.) [28]

*Vigna radiata, Triticum aestivum*	Cell phone, 900 MHz, 4 h exposure	Growth reduction (21 and 50%) in *Vigna* and *Triticum*, respectively [13]

*Triticum aestivum, Cicer arietinum, Vigna radiata, Vigna aconitifolia*	Klystron-based EMF generator, 9.6 GHz, 1 dBm to 3.5 dBm	Growth and biomass reduction [41]

*Vigna radiata, Ipomoea aquatica*	425 MHz, 2 h, 1 mW	Growth stimulation of primary root [16]

*Glycine max*	900 MHz, 5.7 to 41 V m^−1^	Inhibition of epicotyl and/or root growth, depending on exposure set-up [9]

*Lemna minor*	400–1900 MHz, 23 to 390 V m^−1^, whole plant exposure	Growth slowed down, at least in the first days following exposure [18]

*Trigonella foenum-graecum, Pisum sativum*	900 MHz, 0.5–8 h	Increased root size, nodule number, and size [42]

*Hibiscus sabdariffa*	Resulting field from a GSM base antenna (not measured)	Reduction of flower bud abscission with increasing distances from the antenna [43]

*Linum usitatissimum*	Cell phone or Gunn generator (105 GHz), 2 h	Production of epidermic meristems under calcium deprivation condition [44]

*Rosa hybrida*	900 MHz, 5–200 V m^−1^, whole plant exposure in MSRC	Delayed and reduced (45%) growth of secondary axes [45]
